# Current Management of Surgical Oncologic Emergencies

**DOI:** 10.1371/journal.pone.0124641

**Published:** 2015-05-01

**Authors:** Marianne R. F. Bosscher, Barbara L. van Leeuwen, Harald J. Hoekstra

**Affiliations:** Department of Surgical Oncology, University Medical Center Groningen, University of Groningen, Groningen, the Netherlands; School of Medicine, Fu Jen Catholic University, TAIWAN

## Abstract

**Objectives:**

For some oncologic emergencies, surgical interventions are necessary for dissolution or temporary relieve. In the absence of guidelines, the most optimal method for decision making would be in a multidisciplinary cancer conference (MCC). In an acute setting, the opportunity for multidisciplinary discussion is often not available. In this study, the management and short term outcome of patients after surgical oncologic emergency consultation was analyzed.

**Method:**

A prospective registration and follow up of adult patients with surgical oncologic emergencies between 01-11-2013 and 30-04-2014. The follow up period was 30 days.

**Results:**

In total, 207 patients with surgical oncologic emergencies were included. Postoperative wound infections, malignant obstruction, and clinical deterioration due to progressive disease were the most frequent conditions for surgical oncologic emergency consultation. During the follow up period, 40% of patients underwent surgery. The median number of involved medical specialties was two. Only 30% of all patients were discussed in a MCC within 30 days after emergency consultation, and only 41% of the patients who underwent surgery were discussed in a MCC. For 79% of these patients, the surgical procedure was performed before the MCC. Mortality within 30 days was 13%.

**Conclusion:**

In most cases, surgery occurred without discussing the patient in a MCC, regardless of the fact that multiple medical specialties were involved in the treatment process. There is a need for prognostic aids and acute oncology pathways with structural multidisciplinary management. These will provide in faster institution of the most appropriate personalized cancer care, and prevent unnecessary investigations or invasive therapy.

## Introduction

An oncologic emergency is an acute, potentially life threatening condition that has developed directly or indirectly as a result of cancer or cancer treatment [1, 2]. Non-elective consultation for symptoms caused by malignant disease is an important marker of poor prognosis [3–8]. For some oncologic emergencies, surgical interventions may be necessary for dissolution or temporary relieve [9]. Not all cancer patients will benefit from surgery. A surgical intervention is irreversible, and can result in severe complications. For patients with poor performance and/or advanced disease, invasive treatment could have a detrimental impact on the life expectancy and quality of life.

It is hardly possible to draught guidelines for the management of surgical oncologic emergencies. The great inter-patient variability and an even greater variety of influencing factors require that every patient needs to be evaluated individually [9]. In the absence of these guidelines, the most optimal method for objective evaluation and decision making would be discussion in a multidisciplinary cancer conference (MCC) [10]. It is essential to define the prognosis of both the emergency and the cancer stage, and taking into account the patient’s performance score when deciding on the treatment [9, 11]. The most appropriate therapy is the treatment that has clinical benefit, and does not reduce the quality of life. Decisions regarding treatment in emergency situations are often not easy to make, and a multidisciplinary approach can provide in more solid arguments regarding the invasiveness of treatment. In an acute setting, time is scarce and the opportunity for multidisciplinary discussion is often not available. Decisions have to be made timely for prompt management of the emergency, and thus are often made by a single specialist. Acute oncology teams and units have been introduced for the care for patients with oncologic emergencies. These teams could prevent unnecessary investigations or therapy, and can provide in quick referral to palliative care when necessary [12–17]. However, specialized acute oncology care is not widely implemented in common medical practice.

In order to provide arguments for the future development of structural acute oncology pathways for faster institution of optimal care, it is important to be aware of (1) the occurrence of (surgical) oncologic emergencies, (2) the decisional process and the amount of patients being discussed in multidisciplinary cancer conferences, and (3) the clinical outcome of current management. In this study, the management and short term outcome of patients after surgical oncologic emergency consultation was analyzed.

## Materials and Methods

A prospective registration and follow up was performed for adult cancer patients (age > 18) in the University Medical Center Groningen (UMCG), who required consultation for surgical oncologic emergencies, between 01-11-2013 and 30-04-2014. The protocol was consistent with the declaration of Helsinki of 1975, as revised in 1983, and approval for the study was retrieved from the institutional Medical Ethics Committee of the University Medical Center Groningen. Written informed consent was retrieved from participants, and all data were analyzed anonymously.

Criteria for inclusion were: surgical oncologic emergency consultation for symptoms caused by any type of malignant disease (including primary presentation), or for symptoms caused by current or previous cancer treatment (surgery, radiation therapy, chemotherapy, drug targeted therapy). When a surgical oncologist and/or surgical resident was involved in the diagnostic and decisional process, and the possibility of surgical treatment had been evaluated, the consultation was regarded as being a surgical oncologic emergency consultation. Patients who required emergency consultation for symptoms that could not be related to malignant disease or cancer treatment were excluded for analysis. This means that the entire hospital population was studied, including patients who were initially admitted on other than surgical wards (e.g. gynecology, internal medicine) and required surgical oncologic consultation.

Patients who required surgical emergency consultation through four possible pathways were to meet the inclusion criteria: (1) presentation at the Emergency Room (ER), (2) non-elective admission through the (surgical) outpatient clinic, (3) transfer from other hospitals, and (4) in-hospital request of surgical consultation for patients admitted for other specialties.

General patient characteristics were documented upon inclusion; gender, age, oncological history, previous cancer treatment, disease status before the emergency consultation (not being diagnosed with cancer, Alive With Disease—AWD, No Evidence of Disease—NED—after cancer treatment), intention of the current cancer treatment (diagnostic, curative, palliative). The following variables were documented during the follow up: type of surgical oncologic emergency, type of treatment (i.e. surgical procedures or other interventions), number of involved medical specialties during hospital admission, and whether the patient was discussed in a Multidisciplinary Cancer Conference (MCC). In the UMCG, multiple regularly scheduled MCC’s for different cancer types are integrated in common cancer care. In general, they include the disciplines that are involved in the diagnostic process and treatment according to the prevailing guidelines. For this study, a patient was regarded as being discussed in a MCC when a report of the MCC was documented in the patient’s chart.

The follow up period was 30 days. At final follow up, the patients’ charts were analyzed for disease status (AWD, NED), intention of cancer treatment (curative, palliative) and mortality. All data were processed through IBM SPSS Statistics 22 for statistical analysis.

## Results

During the study period, 3737 patients had visited the ER for surgical consultation, and 402 of these patients (11%) had a previous history of cancer, or active malignant disease. After visiting the ER, 147 patients (4% of all 3737 patients, and 37% of the 402 cancer patients) were identified to have surgical oncologic emergencies and were included for analysis. The remaining patients visited the ER for non-oncologic issues.

Further, 19 cancer patients were non-electively admitted through the surgical outpatient clinic for surgical oncologic emergencies, another 35 cancer patients required in-hospital surgical oncologic emergency consultation during admission for other medical specialties, and 6 patients were transferred from other hospitals.

In total, 207 patients with surgical oncologic emergencies were included for analysis through all pathways. There were 101 (49%) males and 106 (51%) females, and median age was 64 (range 19–92) years. Of all patients, 21 patients had a primary presentation of malignant disease, 132 patients were alive with disease (AWD) that was previously diagnosed, and 54 patients had No Evidence of Disease (NED) after being treated for cancer in the past, of whom 9 patients presented with recurrent disease. Of the patients who had been diagnosed with cancer in the past, the most prominent type of cancer was colorectal carcinoma (26%). Table 1 provides an extensive overview of the baseline characteristics for all 207 cancer patients with surgical oncologic emergencies.

**Table 1:** Baseline characteristics of cancer patients who experienced surgical oncologic emergencies.

	Total n = 207
**Male**	101 (48.8)
**Female**	106 (51.2)
**Median Age**	64 (19–92
**ECOG—WHO Performance score**	
0	57 (27.5)
1	85 (41.1)
2	47 (22.7)
3	14 (6.8)
4	4 (1.9)
**ASA classification**	
1	22 (10.6)
2	136 (5.7)
3	49 (23.7)
**Doctors’ shift of consultation**	
Day	126 (60.9)
Evening	24 (11.6)
Night	11 (5.3)
Weekend day	26 (12.6)
Weekend evening/night	20 (9.7)
**Route consultation**	
Emergency Room	147 (71.0)
In-hospital consultation	35 (16.9)
Outpatient clinic	19 (9.2)
Transfer from other hospital	6 (2.9)
**Cancer type**	
Colorectal carcinoma	54 (26.1)
Hepatobiliary	18 (8.7)
Breast cancer	14 (6.8)
Soft tissue sarcoma/GIST	14 (6.8)
Neuroendocrine tumor	13 (6.3)
Melanoma	11 (5.3)
Cervix carcinoma	8 (3.9)
Hematologic malignancy	8 (3.9)
Esophageal carcinoma	7 (3.4)
Non-melanoma skin cancer	6 (2.9)
Lung carcinoma	4 (1.9)
Prostate carcinoma	3 (1.4)
Ovarian carcinoma	3 (1.4)
Gastric carcinoma	2 (1.0)
Other	7 (3.4)
Unknown	14 (6.8)
No cancer	21 (10.1)
**Other type of cancer**	
No	174 (84.1)
Yes	33 (15.9)
**Time since cancer diagnosis**	
No cancer diagnosis before consultation	21 (10.1)
<30 days	26 (12.6)
30 days—6 months	56 (27.1)
6 months—1 year	20 (9.7)
1–2 years	13 (6.3)
2–5 years	41 (19.8)
> 5 years	30 (14.5)
**Documented stage of treatment before surgical oncologic emergency consultation**	
**No cancer**	**21 (10.2)**
**Active disease**	**129 (62.3)**
Diagnostic stage	32 (15.5)
Receiving treatment with curative intent	49 (23.7)
Palliative stage	48 (23.2)
NED* after being treated for cancer in the past	**57 (27.5)**
< 30 days	19 (9.2)
30 days—6 months	10 (4.8)
6 months—1 year	7 (3.4)
1–2 years	6 (2.9)
2–5 years	6 (2.9)
> 5 years	9 (4.3)
**Previous Radiotherapy**	66 (31.9)
**Previous Chemotherapy**	72 (34.8)
**Previous Surgery**	126 (60.9)
**Time since last cancer treatment**	
Continuously	24 (11.6)
< 30 days	62 (30.0)
30 days—6 months	32 (15.5)
6 months—1 year	9 (4.3)
1–2 years	15 (7.2)
2–5 years	5 (2.4)
> 5 years	12 (5.8)
No cancer treatment	48 (23.2)

* NED: No Evidence of Disease

### Baseline characteristics of cancer patients who were consultated for surgical oncologic emergencies

Obstruction (e.g. colorectal, biliary, small intestine), and infection were the most frequent conditions for surgical oncologic emergency consultation (42% and 32% respectively) (Table 2).

**Table 2:** Diagnosis after surgical oncologic emergency consultation and 30 day follow up for surgical interventions, mortality, and discussion in a multidisciplinary cancer conference (MCC) within the follow up period.

Classification	N	Diagnose		N	Surgery	Deceased	MCC*
**Obstruction**	86	**Malignant**		**62**	**38 (61.3)**	**8 (12.9)**	**26 (41.9)**
			Colorectal	22	16 (72.7)	3 (13.6)	11 (50.0)
			Biliary	19	7 (36.8)	1 (5.3)	6 (31.6)
			Small intestine	18	14 (77.8)	4 (22.2)	7 (38.9)
			Airway	2	1 (50.0)	-	2 (100)
			Gastroesofageal	1	-	1 (100)	-
		**Benign**		**24**	**10 (41.7)**	**1 (4.2)**	**4 (16.7)**
			Colorectal	8	1 (12.5)	-	1 (12.5)
			Small intestine	7	5 (71.4)	1 (14.3)	1 (14.3)
			Radiation enteritis	4	4 (100)	-	1 (25.0)
			Biliary	3	-	-	1 (33.3)
			Gastroesofageal	1	-	-	-
			Urinary	1	-	-	-
**Infection**	67	**Postoperative wound infection**		**25**	**3 (12.0)**	**2 (8.0)**	**5 (20.0)**
			Score 1 or 2**	6	-	-	-
			Score 3 or 4	17	2 (11.7)	-	5 (29.4)
			Score 5	2	1 (50.0)	2 (100)	-
		**Infection/neutropenic enterocolitis during chemotherapy**		**11**	**4 (36.4)**	**2 (18.2)**	**-**
		**Fistula formation after surgery**		**7**	**2 (28.6)**	**-**	**-**
		**Intraabdominal infection after surgery**		**7**	**1 (14.2)**	**-**	**3 (42.9)**
		**Infectious tumor mass**		**5**	**3 (60.0)**	**1 (20.0)**	**3 (60.0)**
		**Wound healing disturbance after radiation therapy and Surgery**		**4**	**1 (25.0)**	**-**	**-**
			Score 1 or 2**	3	1 (33.3)	-	-
			Score 3	1	-	-	-
		**Chronic presacral absess formation after pelvic surgery and radiation therapy**		**3**	**-**	**-**	**1 (33.3)**
		**Postoperative gastroenteritis**		**3**	**-**	**-**	**-**
		**Lymphedema/erysipelas**		**2**	-	**-**	-
**Clinical deterioration**	19	**Clinical deterioration due to progressive metastatic disease**		**9**	**1 (11.1)**	**4 (44.4)**	**2 (22.2)**
		**Clinical deterioration and pain due to progressive tumor mass**		**8**	**3 (37.5)**	**3 (37.5)**	**4 (50.0)**
		**Clinical deterioration being NED** ***		**2**	**-**	**1 (50.0)**	**-**
**Gastrointestinal leak**	12	**Perforation in the presence of tumor mass**		**7**	**6 (85.7)**	**3 (42.9)**	**1 (14.3)**
		**Anastomotic leak after surgery**		**5**	**3 (60.0)**	**-**	**2 (40.0)**
**Bleeding/thrombosis**	12	**Tumorbleeding**		**8**	**2 (25.0)**	**-**	**3 (37.5)**
		**Paraneoplastic arterial/venous thrombosis**		**3**	**1 (33.3)**	**-**	**1 (33.3)**
		**Postoperative bleeding**		**1**	**1 (100)**	**-**	**1 (100)**
**Pathological fracture**	5	**Fractures due to bone metastases**		**5**	**3 (60.0)**	**-**	**1 (20.0)**
**Other**	6	**Lymphadenopathy/malignant swelling**		**3**	**1 (33.3)**	**-**	**1 (33.3)**
		**Chylus leakage postoperative**		**2**	**-**	**-**	**1 (50.0)**
		**Incidental diagnosis on imaging studies**		**1**	**-**	**-**	**1 (100)**

* **MCC: Multidisciplinary Cancer Conference**

** **According to the Southampton Wound Assesment Scale**

*** NED: No Evidence of Disease

After surgical oncologic emergency consultation at the ER, 109 of the 147 patients (74%) were directly hospitalized. Four of the remaining 38 patients (11%) had an emergency admission within 30 days after the first consultation at the ER. Together with the patients who were already hospitalized before the surgical oncologic emergency consultation (the patients who required in-hospital consultation or transfer from other hospitals), 173 of all patients with surgical oncologic emergencies (84%) had been hospitalized during the study period.

During hospitalization, the median number of radiologic, endoscopic, and surgical interventions was 1 (range 0–09). Eighty three of all patients (40%) underwent surgery during the follow up period. The median duration between inclusion and surgery was 38 hours (range 0–720 hours/30 days). Of these patients, 70 patients (84%) underwent surgery in an emergency setting after a median period of 25.5 (range 0–720) hours, and 13 patients (16%) underwent elective surgical procedures after a median period of 16 (range 7–30) days.

The median number of involved medical specialties during admission was 2 (range 1–8). Within 30 days after surgical oncologic emergency consultation, 61 patients (30%) were discussed in a MCC, after a median duration of 12 (range 1–30) days. For only 25 of these patients (15% of all hospitalized patients, and 41% of all patients who were discussed), the MCC took place while they were hospitalized after a median period of 8 (range 1–35) days after emergency consultation. The remaining 36 patients were discussed in a MCC after discharge from the ER or hospital ward. Of the 62 patients with symptoms caused by malignant obstruction, 42% were discussed in a MCC (Table 2), and 61% of these patients underwent surgical treatment during the follow up period. Gastrointestinal perforation in the presence of tumor mass (14%), benign obstruction (17%), and postoperative wound infections (20%) were the diagnoses with the lowest rates of multidisciplinary discussion.

**Fig 1:**
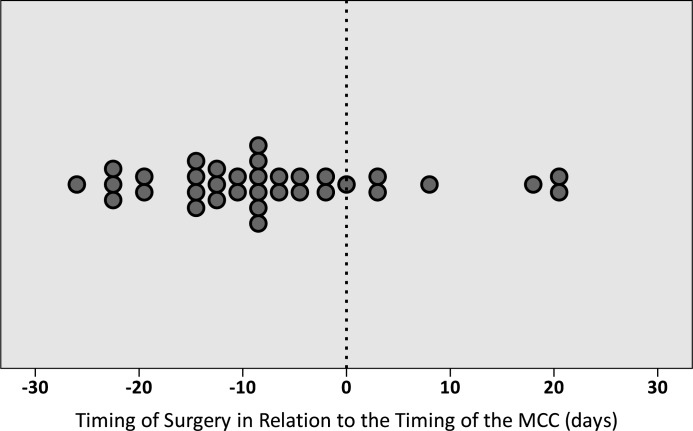
The timing of surgery in relation to the timing of the Multidisciplinary Cancer Conference (MCC). The MCC is set as timepoint 0.

Only 34 (41%) of the 83 patients who underwent surgery were discussed in a MCC during the follow up period. For 27 of these 34 patients (79%), the surgical procedure was performed before the MCC, and only 7 patients (21%) were discussed in a MCC prior to surgery. Regarding the moment of surgery in relation to the moment of the MCC, the median period was 9 days prior to (range 26 days prior to—21 days after) the MCC (Fig 1).

Before surgical oncologic emergency consultation, 32 patients (16%) were in a diagnostic and/or staging process, 49 patients (24%) received cancer treatment with curative intent, 57 patients (28%) had NED after being treated for cancer in the past, and 48 patients (23%) were diagnosed to have incurable malignant disease and were in a palliative stage of treatment. Another 21 patients (10%) had no cancer diagnosis before surgical oncologic emergency consultation, and had a primary presentation of malignant disease. At final follow up, 70 patients (34%) received adjuvant treatment with curative intent or were scheduled for supplementary curative surgical procedures, 42 patients (20%) were NED, and 69 patients (33%) were in a palliative stage, and 26 patients (13%) were deceased.

**Fig 2:**
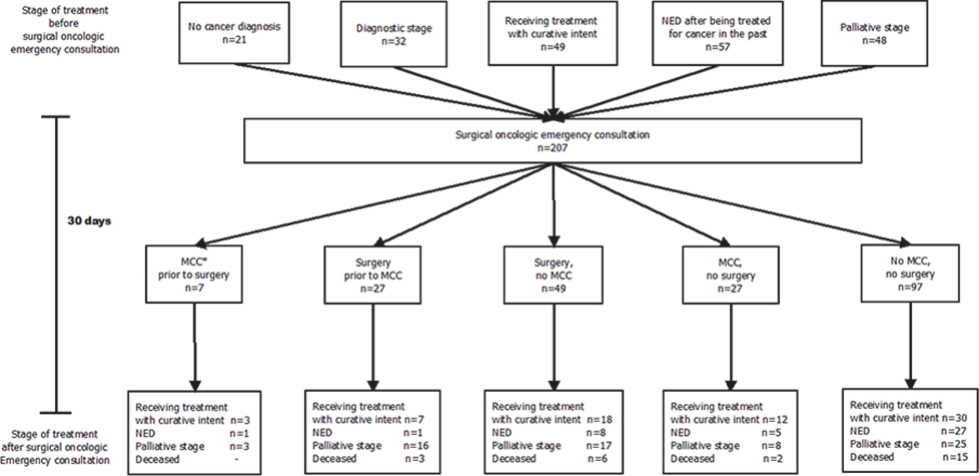
The 30-day clinical pathway of cancer patients after surgical oncologic emergency consultation. Starting at stage of treatment prior to the consultation, whether patients undergo surgery and/or are being discussed in a *Multidisciplinary Cancer Conference (MCC), and stage of treatment 30 days after surgical oncologic emergency consultation.

Many of the patients who were in a palliative stage at final follow up had undergone surgery after inclusion (52%), and 35% of all the patients who were deceased. Most patients died of progressive disease (77%) and 23% died of clinical sepsis or multiple organ failure. Of the deceased patients, 12 (46%) died at home after the institution of palliative care, 10 (39%) died during hospital admission, and 4 patients (15%) were transferred to a nursing home or hospice. Fig 2 visualizes the clinical pathway of the cancer patients in this study.

## Discussion

To our knowledge, this is the first extensive analysis of surgical oncologic emergencies and the management in clinical practice. For 37% of the cancer patients who had visited the ER, the surgical consultation at the ER was related to a surgical oncologic emergency. Surgeons will not only be confronted with oncologic emergencies through the ER, but also through the outpatient clinic, and in- or inter-hospital consultation. Almost a third of the patients in this cohort were consultated through other pathways than the ER.

In the past decades, MCCs have become common practice, especially in elective oncology care [18]. Cancer patients represent a complex population and often require treatment from multiple medical specialties. In this study, only 30% of the patients who had been consultated for surgical oncologic emergencies had been discussed in a MCC within 30 days after emergency consultation. This is strikingly low, since the national and institutional guidelines require that every cancer patient is discussed in a MCC to establish general agreement before the start of cancer treatment. For all 33 patients the MCC took place at a regular weekly schedule, and acute multidisciplinary discussion upon admission was not available. This means that for the majority (79%) of the patients who were discussed, emergency treatment was instigated before the MCC; for the 34 patients who underwent surgery and who had been discussed, there was a median period of -9 days in relation to the MCC. The rate of patients being discussed in a MCC was regardless of the amount of medical specialties that were involved during admission (a median of 2 different specialties per patient).

These results confirm the outcome of other studies, that for the most cancer patients who are non-electively treated for surgical oncologic emergencies, emergency (surgical) management—or the decision to refrain from surgery—is performed without discussing the patient in a MCC [10]. Physicians of different medical specialties, who are involved in the treatment process of one patient, can have one-to-one transmissions regarding field specific issues of attention. Nevertheless, without discussing these issues in an organized group-setting, no overall objective view will be obtained in order to connect all issues and transfer these into the same direction of treatment. For patients who require emergency treatment,—non-scheduled—multidisciplinary evaluation by acute oncology experts should be available.

Obstruction is the most frequent oncologic emergency seen in surgical practice [9]. In this study, of all patients with surgical oncologic emergencies, 42% had symptoms of obstruction with either malignant or benign origin. Surgery often seems to be the best solution for relieve of the obstruction, but could also have an adverse influence on the survival and quality of life. Cancer stage and the performance status of the patient are the most important predictors of survival, and the main factors to influence the successfulness of invasive therapies [11, 19–21]. Patients with obstruction of the gastrointestinal tract often require emergency surgery, and the time frame until the next scheduled MCC will be too large. For all oncologic emergencies, evaluation of all treatment options is essential. Even if the consequences of the emergency are fatally, the quality of life remains the highest priority at the end of life. Only 42% of the 62 patients with symptoms caused by malignant obstruction were discussed in a MCC. However, 61% of all these patients underwent surgical treatment. Gastrointestinal perforation in the presence of tumor mass, benign obstruction, and postoperative wound infections were the diagnoses of patients with the lowest rate of multidisciplinary discussion. The severity of diagnoses (wound infection), and time (gastrointestinal perforation) are possibly factors that have had influence on the different rates of multidisciplinary management.

The number of patients with poor outcome after surgical oncologic emergency consultation was high. Within 30 days, 33% of patients had ended in a palliative stage and 13% were deceased. Taken together, 46% of all patients had poor outcome on very short term. This was twice as many compared to the 23% of patients who were already in a palliative (and thus poor) stage before inclusion. Other studies have reported 30-day mortality rates of 10% and 30% after emergency abdominal surgery in cancer patients [11, 22]. The cohort of patients in this study represents a more heterogeneous category, however, the 30-day mortality rate remains high. Regardless of the outcome, many patients had undergone surgery. Of the patients who ended in a palliative stage, 52% had undergone surgery during the study period, and 35% of all the patients who were deceased.

Physicians have the tendency to overestimate the life expectancy of terminally ill cancer patients, and it is against the nature of many to spare someone from treatment [23–25]. An earlier study by Ramchandran et al. tried to create a prediction model to identify hospitalized cancer patients at risk for 30-day mortality, based on information only from the electronic medical record [26]. Patients’ performance scores were not included in the model, because it requires clinical assessment of the patient. However, the performance score has been reported to be one of the most important predictors of outcome [19–21, 27]. Further research to identify influencing factors, and the development of prognostic tools, is necessary for more accurate prediction of outcome in the acute setting. Prognostic aids for decision making in a multidisciplinary setting will contribute to argumentation for (refraining from) invasive therapies. Further, when the expected outcome of therapies, or a near death, is communicated to the patient and family, it can prevent disappointment after non-successful invasive treatment, and preserve the quality of a patient’s life during the last stage [28, 29].

The heterogeneity of the common cancer patient population, and the variety of surgical oncologic emergencies is evident in this study. The interpatient variety (patient performance, cancer stage) is the cause of variable clinical outcome and impedes guidelines for management of these emergencies. This heterogeneity is the core of the difficulties and dilemmas in clinical (surgical) practice, and supports the need for the development of decision aids and acute oncology pathways with structural multidisciplinary management.

Since this is an observational study, it is not possible to evaluate if the treatment of patients with surgical oncologic emergencies would have been different when the decisional process had involved a MCC. The reasons why some patients were discussed in a MCC and others were not is not recorded in this study. For patients who were discussed and underwent surgical procedures, the median time period of 9 days between surgery and a MCC implicates that at this point the MCC’s are used for decision making after a pathology result is present, and not for acute treatment decisions including surgery. Furthermore, the fact that, also for many patients who were not discussed in a MCC, multiple medical specialties were involved in the treatment process, could reflect the complexity of pathology.

This study was performed in one tertiary university hospital, and comparison to other hospitals will be difficult. However, since the patient population represents an entire hospital population, the authors believe that the results of the current study reflect common medical practice. In most hospitals, patients with oncologic emergencies will present through the ER, and specialized acute oncology care has not been implemented in standard emergency care.

The implementation of acute oncology pathways, providing systematic multidisciplinary management of all patients, would be the most optimal way for decision making and treatment of patients with oncologic emergencies [12–17]. Acute oncology care should include structural availability of a specialized team of (at least) an emergency care specialist, a surgical oncologist, a radiation oncologist, a medical oncologist, a palliative care specialist, and an oncology nurse. This team will be trained in acute oncology care, and should be available throughout the day and evening (in exclusive cases during the night). The members of this acute oncology team need to be involved in the evaluation and treatment process directly after emergency presentation. In this way, non-scheduled multidisciplinary decision making will be possible and personalized treatment can be instituted on the shortest term, preventing delay of required therapies or overtreatment.

Close involvement of the patient’s general practitioner is required during hospital admission. In this way, when invasive treatment is not expected to be favorable for the patient, palliative care can be instituted more efficiently and on shorter term. At the end of life, the length of hospitalization should be limited to only what is needed for care with clinical benefit.

Further prospective research is necessary to investigate the influence of acute oncology pathways and structural multidisciplinary management on the clinical outcome and quality of life.

## Conclusions

Obstruction (i.e. colorectal, biliary, small intestine) and infection were the most frequent conditions for surgical oncologic emergency consultation. Many patients ended in a palliative stage, and the overall mortality within 30 days was 13%. In most cases, emergency treatment, including invasive therapies such as surgery, occurred without discussing the patient in multidisciplinary cancer conferences, regardless of the fact that multiple medical specialties were involved in the treatment process. There is a need for the development and evaluation of prognostic aids and acute oncology pathways providing in structural multidisciplinary management. It will result in institution of the most appropriate personalized cancer care on the shortest term, preventing delay of required therapies or overtreatment.
